# Observations on Side-Swimming Rainbow Trout in Water Recirculation Aquaculture Systems

**DOI:** 10.1080/08997659.2014.938870

**Published:** 2014-09-17

**Authors:** Christopher Good, John Davidson, Christin Kinman, P. Brett Kenney, Grete Bæverfjord, Steven Summerfelt

**Affiliations:** ^a^ The Conservation Fund's Freshwater Institute, 1098 Turner Road, Shepherdstown, West Virginia25443, USA; ^b^ Division of Animal and Nutritional Sciences, West Virginia University, Morgantown, West Virginia26506-6108, USA; ^c^ Nofima Marin, SjølsengN-6600, Sunndalsøra, Norway

## Abstract

During a controlled 6-month study using six replicated water recirculation aquaculture systems (WRASs), it was observed that Rainbow Trout *Oncorhynchus mykiss* in all WRASs exhibited a higher-than-normal prevalence of side swimming (i.e., controlled, forward swimming but with misaligned orientation such that the fish's sagittal axis is approximately parallel to the horizontal plane). To further our understanding of this abnormality, a substudy was conducted wherein side swimmers and normally swimming fish were selectively sampled from each WRAS and growth performance (length, weight), processing attributes (fillet yield, visceral index, ventrum [i.e., thickness of the ventral “belly flap”] index), blood gas and chemistry parameters, and swim bladder morphology and positioning were compared. Side swimmers were found to be significantly smaller in length and weight and had less fillet yield but higher ventrum indices. Whole-blood analyses demonstrated that, among other things, side swimmers had significantly lower whole-blood pH and higher Pco_2_. Side swimmers typically exhibited swim bladder malformations, although the positive predictive value of this subjective assessment was only 73%. Overall, this study found several anatomical and physiological differences between side-swimming and normally swimming Rainbow Trout. Given the reduced weight and fillet yield of market-age side swimmers, producers would benefit from additional research to reduce side-swimming prevalence in their fish stocks.

Received March 20, 2014; accepted May 20, 2014

Side swimming is an understudied anomaly occasionally exhibited by various fish species under intensive culture conditions (Branson and Turnbull 2008). Side swimmers swim in a coordinated manner but are oriented abnormally in the water column (Figure 1), such that their dorsal and ventral aspects are approximately perpendicular to the direction of gravity. Despite the change in orientation, side swimmers appear to consume feed well and grow similarly to normally swimming fish. The authors have observed side-swimming fish in a variety of aquaculture settings, including flow-through raceways, circular tanks in water recirculation aquaculture systems (WRASs), and freshwater net-pens. The authors are familiar with several commonly held beliefs concerning the reason(s) for side swimming, including swim bladder malformation, increased intracoelomic adipose tissue, increased adipose tissue deposition in the ventral connective tissue (i.e., the “belly-flap”), and prolonged exposure to rotational water currents in circular tanks; however, very little scientific research has been carried out to investigate these theories or to characterize the basic differences between side-swimming and normally swimming fish. In one of the few studies documenting side swimming, Davidson et al. (2011a) found that side swimming was exacerbated in Rainbow Trout *Oncorhynchus mykiss* cultured in WRASs with low water exchange rates and identified accumulating concentrations of nitrate nitrogen (NO_3_-N) and dissolved potassium as potential causal factors; further controlled research (Davidson et al. 2014) found a significant association between Rainbow Trout side swimming and elevated NO_3_-N.

**FIGURE 1.**
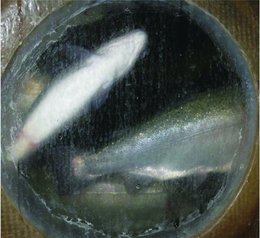
View from a circular tank side window illustrating both side-swimming (top) and normally swimming (bottom) Rainbow Trout. [Color figure available online.]

An important consideration for producers is whether side swimming is actually related to reduced fish performance, i.e., whether reducing side swimming in fish populations would lead to increased growth, survival, fillet yield, or overall farm profitability. Additionally, in the authors’ experience side swimmers are inherently conspicuous and are routinely commented upon by members of the public touring aquaculture facilities; therefore, side swimming might contribute to negative perceptions regarding the health, welfare, and product quality of farmed fish.

A controlled 6-month study using six replicated WRASs was carried out examining Rainbow Trout performance in low-water-exchange, ozonated systems versus high-water-exchange, nonozonated systems (Davidson et al. 2011b). Approximately midway through this experiment, it was noted that a higher-than-normal prevalence of Rainbow Trout were exhibiting side swimming in all WRASs, with the prevalence of side swimming being significantly higher in low-exchange WRASs (Davidson et al. 2011a). In light of this unexpected finding, a substudy was planned to investigate side swimmers at the end of the original experiment when the fish were at, or slightly larger than, market size. The objective of this study was to characterize anatomical and physiological differences between side-swimming and normally swimming fish, in particular to determine whether side swimming was associated with reduced performance or product yield.

## METHODS

### Water recirculation aquaculture systems

The experimental WRASs used during the present study have been previously described by Davidson et al. (2009), and the specific operation of these systems for the initial controlled experiment was described in detail by Davidson et al. (2011b). Briefly, the study used six identical 9.5-m^3^ WRASs with 5.3-m^3^ circular dual-drain tanks, fluidized sand biofilters, and low-head oxygenators that provided rearing-unit dissolved oxygen at approximately saturation. Three WRASs were operated at low exchange (0.26% makeup water relative to a total recirculation flow of 380 L/min) and received water ozonation via generators (model G22; Pacific Ozone Technology, Benecia, California) that converted a portion of pure-oxygen feed gas to ozone, which was then transferred to water within the systems’ low-head oxygenators. An SC100 Universal Controller (Hach) provided proportional-integral-derivative control of generator output to maintain ozone oxidation–reduction potential at a set point of 250 mV, which ensured that ozone dosage was approximately 20–25 g ozone/kg feed throughout the study. The remaining three WRASs were operated at high exchange (2.6% makeup water relative to the total recirculation flow) and did not receive water ozonation.

### Rainbow Trout

The Rainbow Trout used in this experiment were acquired as eyed eggs from a commercial producer and raised on site, initially as fry in small (0.5-m^3^) flow-through circular tanks. Fish were stocked into the replicated WRASs (1,000 fish/system) at a mean ± SE size of 151 ± 3 g and were maintained for 6 months at a density of 30–80 kg/m^3^. A constant 24-h photoperiod was provided, and feed was administered every alternate hour (with two feed events occurring within that hour) using automated feeders (T-drum 2000CE; Arvo-Tec, Finland). Feeding rates were based on standardized feeding charts but were modified occasionally according to feeding activity and observable wasted feed. A slow-sinking trout feed (42:16 protein-to-fat ratio; Zeigler Brothers, Gardners, Pennsylvania) was used throughout the study.

### Fish sampling

At the end of the study (370 d posthatch, overall average fish size = 1,364 g), three side swimmers and three normally swimming Rainbow Trout from each WRAS (36 fish in total) were selectively sampled and sequentially euthanized with an overdose (200 mg/L) of tricaine methanesulfonate (MS-222; Western Chemical, Ferndale, Washington). Fish were individually brought to a team of researchers who were blinded as to whether each fish was a side swimmer or a normal swimmer. Fish were first bled via caudal venipuncture using a 21-guage, 38-mm needle and 3-mL syringe. Whole-blood samples were immediately analyzed using an i-Stat 1 portable analyzer (Abbott Laboratories, Abbott Park, Illinois) with CG4+ (measuring Pco_2_ [partial pressure of the gas], Po_2_, HCO_3_, total CO_2_, O_2_ saturation, and lactate) and CHEM8+ (measuring sodium, potassium, chloride, calcium, glucose, creatinine, hematocrit, and hemoglobin) cartridges. Fish were measured for length and weight, then the viscera was removed through a ventral incision, while taking care not to disturb the position or integrity of the swim bladder. With the viscera removed, photographs were taken of each swim bladder in situ (32 swim bladder photographs were obtained overall). Fish were then butterfly filleted, the fillets were weighed, and measurements were taken for fillet thickness and ventral “belly-flap” thickness using digital microcalipers. These measurements were taken at three locations along the length of the carcass, at approximately the 5th (A), 50th (B), and 95th (C) percentile of the overall fillet or belly-flap length (cranial to caudal). Lastly, at a later date swim bladder photographs were independently assessed by three on-site researchers with advanced experience in fish carcass processing and were categorized as normal or abnormal based on the swim bladder position, level of inflation, or general morphology.

### Calculations and statistical analyses

Total fillet yield for each fish was calculated by dividing the weight of both right and left fillets by fish weight. Percentage viscera was likewise calculated as the visceral weight divided by the fish weight. Ventrum indices were calculated at points A, B, and C by dividing the belly-flap width by fish weight, and fillet thickness indices were calculated at the three points by dividing the thickness of the right fillet by the sum of the right and left fillet thicknesses.

Linear regression models were run on each of the fish-processing metrics listed above and on the individual blood chemistry parameters using SPSS (IBM, Armonk, New York). For each regression model, swimming status served as the independent variable with “tank” added as a covariate to control for the effects of both rearing-unit clustering and treatment effects (i.e., low exchange, ozone versus high exchange, no ozone) from the original controlled study. Individual blood chemistry parameters and processing metrics served as dependent variables.

To assess whether swim bladder categorization (i.e., normal or abnormal) was associated with side-swimming fish, positive and negative predictive values (PPVs and NPVs, respectively) (Szklo and Nieto 2004; Duffy et al. 2005) were calculated based on the results from the three raters. Given the cell identifiers (a through d) listed in Table 1, which identify the four status (side swimmer or normal swimmer) and swim bladder rating (normal or abnormal) combinations, the PPV and NPV were calculated as follows:

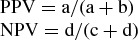



The sensitivity and specificity (Altman and Bland 1994a) of this visual assessment was calculated by the following:

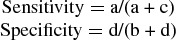

**TABLE 1.** Summary of swim bladder (*n* = 32) assessments by three blinded raters compared with the actual status of fish as side swimmers or normal swimmers. The letters in parentheses (a–d) identify the four status and rating combinations and correspond to formulas for positive and negative predictive values, sensitivity, and specificity, as listed in the Methods section.

	Actual status		
Swim bladder rating and total	Side swimmer	Normal swimmer	Total
Abnormal	47 (a)	17 (b)	64
Normal	1 (c)	31 (d)	32
Total	48	48	96

A two-way mixed-effects model, type absolute agreement, single-measures intraclass correlation (Norman and Streiner 2008) was calculated to determine the scoring consistency between raters.

## RESULTS

**FIGURE 2.**
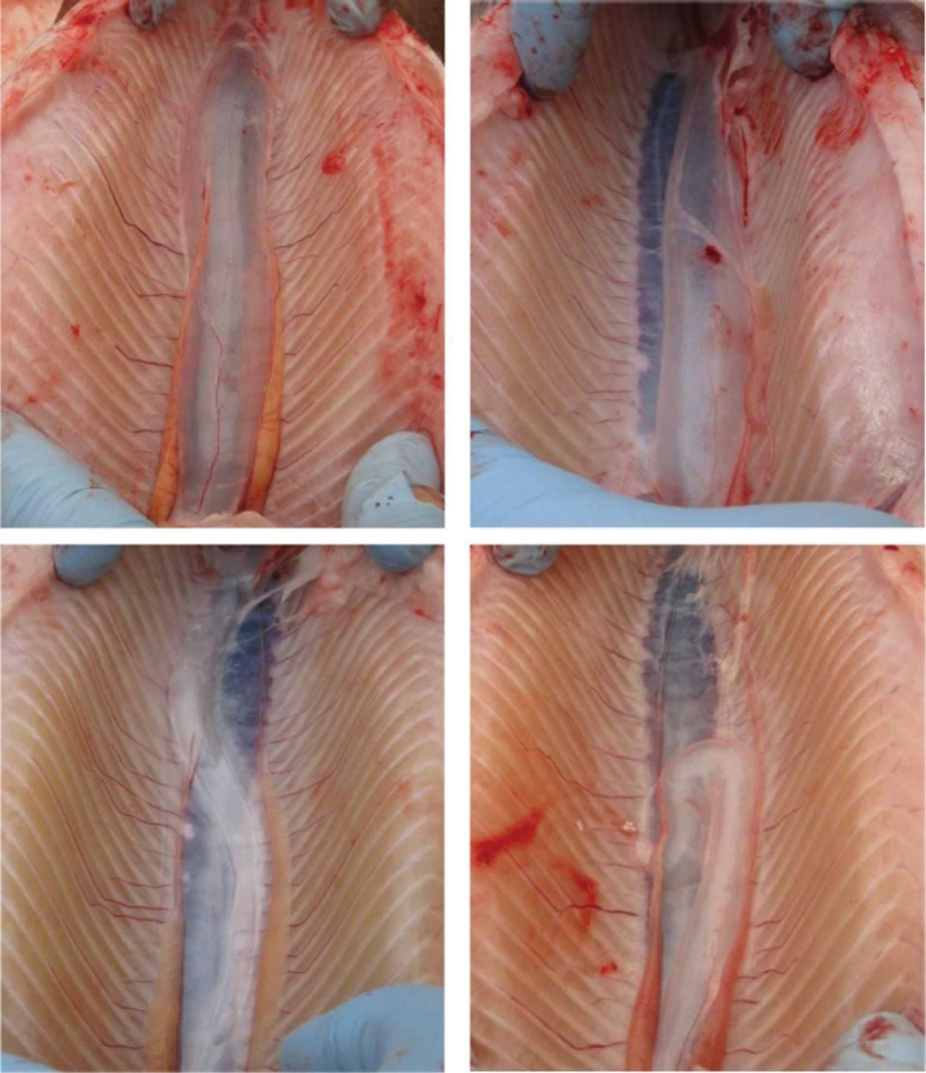
Photographs of eviscerated Rainbow Trout (cranial = bottom of photo), demonstrating qualitative swim bladder abnormalities observed during sampling. The panels are as follows: top left = normal inflation and position; top right = normal inflation, lateral deviation; bottom left = under inflation, s-shaped deviation; and bottom right = under inflation, tortuous malformation, and cranial retroflexion. [Color figure available online.]

Swim bladders that were visually assessed ranged from normal (typical morphology, positioning, and inflation level) to having a variety of malformations, some of which are illustrated in Figure 2. In determining the reliability of the visual swim bladder assessment, the PPV of the test was 73%, with a NPV of 97%, and the test sensitivity and specificity were 98% and 65%, respectively. The intraclass correlation was determined to be 0.772, and there were no significant differences between the raters in their responses determining normal and abnormal swim bladders. Visual swim bladder assessment results are summarized in Table 1.

Linear regression modeling of blood chemistry parameters revealed significant (*P* < 0.05) differences in potassium, glucose, pH, lactate, and Pco_2_ between side swimmers and normally swimming fish (Table 2). In side swimmers, whole-blood potassium, pH, and lactate were significantly lower, while glucose and Pco_2_ were significantly higher.

**TABLE 2.** Whole-blood gas and chemistry analysis results (mean ± SE) for side-swimming and normally swimming Rainbow Trout. Parameter values that are significantly different (*P* < 0.05) between side swimmers and normal swimmers are indicated in bold italics.

Parameter	Side swimmers	Normal swimmers
Sodium (mmol/L)	147.0 ± 0.5	148.3 ± 0.7
Potassium (mmol/L)	***2.772*** ± ***0.060***	***3.106*** ± ***0.116***
Chloride (mmol/L)	134.1 ± 0.4	134.9 ± 0.5
Calcium (mmol/L)	1.454 ± 0.019	1.483 ± 0.015
Glucose (mg/dL)	***61.78*** ± ***1.18***	***58.06*** ± ***1.50***
Urea nitrogen (mg/dL)	9.056 ± 1.116	10.83 ± 1.33
Hematocrit (% PCV^a^)	28.56 ± 1.01	25.94 ± 0.10
Hemoglobin (g/dL)	9.711 ± 0.345	8.828 ± 0.340
pH	***7.080*** ± ***0.012***	***7.135*** ± ***0.013***
Total CO_2_ (mmol/L)	11.11 ± 0.36	10.65 ± 0.38
Pco_2_ (mm Hg)	***34.27*** ± ***1.79***	***29.18*** ± ***1.18***
Po_2_ (mm Hg)	21.72 ± 1.12	25.82 ± 2.84
O_2_ saturation (%)	22.61 ± 2.20	33.00 ± 5.19
HCO_3_ (mmol/L)	10.02 ± 0.30	9.771 ± 0.335
Lactate (mmol/L)	***0.8822*** ± ***0.0845***	***1.215*** ± ***0.100***

^a^ Percent packed-cell volume.

**TABLE 3.** Summary of processing attributes (mean ± SE) for side-swimming and normally swimming Rainbow Trout. Measurements for the fillet thickness index and the ventrum index (ventral belly-flap thickness) were taken at three locations along the length of the carcass, at approximately the 5th (A), 50th (B), and 95th (C) percentile of the overall fillet or belly-flap length. Parameter values that are significantly different (*P* < 0.05) between side swimmers and normal swimmers are indicated in bold italics.

Parameter	Side swimmers	Normal swimmers
Length (mm)	***389.7*** ± ***5.2***	***412.7*** ± ***3.7***
Weight (g)	***1,139*** ± ***45***	***1,394*** ± ***34***
Ventrum index (A)	0.00898 ± 0.00026	0.00859 ± 0.00027
Ventrum index (B)	***0.01081*** ± ***0.00042***	***0.00973*** ± ***0.00033***
Ventrum index (C)	***0.01090*** ± ***0.00040***	***0.00934*** ± ***0.00026***
Right fillet thickness index (A)	***0.4773*** ± ***0.0032***	***0.4880*** ± ***0.0020***
Right fillet thickness index (B)	0.5041 ± 0.0019	0.5060 ± 0.0018
Right fillet thickness index (C)	0.5163 ± 0.0032	0.5094 ± 0.0029
Total fillet yield (%)	***48.50*** ± ***0.42***	***50.67*** ± ***0.46***
Viscera (%)	15.52 ± 0.56	14.91 ± 0.48

Regression analyses also revealed significant differences in performance and processing metrics between side swimmers and normal swimmers (Table 3). Overall, side swimmers were significantly smaller in both length and weight and had lower fillet yields than normally swimming fish. As well, side swimmers had significantly thicker ventrum indices at points B and C, and their right fillet index at point A was also significantly lower.

## DISCUSSION

The results of this study suggest that, despite appearing otherwise healthy and feeding well, side-swimming Rainbow Trout do not perform as well, in terms of growth and fillet yield, as their normally swimming conspecifics. These differences appear to be subtle, albeit statistically significant, as side swimmers in general are mostly indistinguishable from normal swimmers once they are removed from the water and their behavior can no longer be observed. It is likely that the small but significant differences in size and fillet yield of side swimmers can be attributed to less efficient swimming and an inherent stress associated with swimming in a different orientation than the majority of fish in a rearing unit, as is evidenced by differences in whole-blood parameters between the two types of swimmers. Increased blood carbon dioxide (Pco_2_) in side swimmers implies a relatively higher level of muscular exertion, which corresponds with observations that side swimmers tend to have an increased frequency of body movement despite moving forward in the water column at approximately the same speed as normally swimming fish. The higher Pco_2,_ in turn, appears to have affected other blood parameters in side-swimming fish, such as the lower pH due to CO_2_-associated acidosis and lower Po_2_ and O_2_ saturation due to the resultant Bohr and Root effects of low blood pH (Wedemeyer 1996). Blood glucose was also relatively elevated in side-swimming fish, indicating either higher exertion in swimming, stress, or both. However, a corresponding increase in blood lactate, as would be expected in fish with relatively increased exercise or higher levels of stress, was not noted in side-swimming fish; in fact, side swimmers had statistically lower levels of blood lactate than normally swimming fish. This apparent inconsistency requires further research for adequate explanation, including assessments of blood cortisol and time-to-exhaustion swimming exercise studies. At present, it is clear from the data obtained in this study that alterations in blood chemistry and gas physiology are associated with side swimming, and it is likely that these changes, in the long term, have affected growth and muscle yield. Further research is necessary to determine the economic impact of increased side swimming in farmed fish populations.

Anecdotal information from producers has suggested that increased adipose deposition in the ventral connective tissue (i.e., the belly flap) can lead to disruption in normal buoyancy and subsequent abnormalities in swimming, including side swimming. Ventral indices calculated in this study support the notion that side swimmers have relatively greater belly flap thicknesses, and therefore it is possible that buoyancy is disrupted in side swimmers due to morphological differences in the ventral body wall. However, despite being statistically different, the empirical values of the ventrum indices were not overly different between the two swimming types, and therefore it is likely that increased belly flap thickness is, at best, only a contributing factor in the development of abnormal swimming. This latter notion is supported by the more compelling evidence obtained in this study that swim bladder malformation has a relatively strong association with side swimming.

Swim bladder malformation most likely occurs in the early stages of anatomical development (Poppe et al. 1997); because the Rainbow Trout used in the present study were raised as a single cohort prior to study commencement, any environmental conditions promoting swim bladder malformation were common for all individuals regardless of their subsequent experimental treatment assignment. We report a strong correlation between malformed swim bladders (subjectively determined to be abnormal in morphology or position) and side swimming, based on the strong PPV of the subjective assessment. Predictive values are normally used to judge the usefulness of a diagnostic test (Altman and Bland 1994b) but are limited in that they are, in part, determined by the prevalence of disease in a population, which can change over time. In the present study, pretest probability was set at 50% (i.e., an equal number of side swimmers and normally swimming fish were selectively sampled), and therefore the effect of side-swimming prevalence change in a population on the predictive values of the visual test was not assessed. However, as the visual assessment of swim bladders is, by nature, a lethal test, predictive value calculations carried out in this study were not intended to determine the usefulness of this test for “diagnostic” purposes. Instead, these values were calculated to determine the association of swim bladder malformation with side swimming in light of the fact that swim bladder assessment is an inherently subjective test. It is interesting to note that almost all the assessments of true side swimmers indicated abnormal swim bladders, while a sizeable proportion of normal swimmers also had swim bladders assessed as abnormal. The very high NPV indicates that swim bladder malformation may be a necessary component of the side-swimming risk factor constellation; however, from the lower PPV it is clear that certain fish are still able to swim normally despite possessing a malformed swim bladder and, hence, are not influenced by additional risk factors that would be sufficient to induce side swimming. From these results, it appears that side swimming is a complex, multifactorial abnormality, and further research to understand the factors associated with this condition, particularly prospective study in which causation can be more appropriately assessed, is required.

Overall, the results of this study indicate that, due to the reduced performance of side swimmers relative to normally swimming fish, successful efforts to reduce side swimming in farmed fish populations could lead to increased farm profitability. Further research is required to understand this phenomenon and its genesis in Rainbow Trout and in other important farmed fish species, as the causes of side swimming are still unknown and most likely vary from one species to another. Given the association between swim bladder malformation and side swimming determined in this study, it is important to fully understand the mechanisms and timing of swim bladder formation and inflation in Rainbow Trout to reduce malformation prevalence or to aid in the separation of fish capable of optimal growth from those without functional swim bladders. Better and earlier diagnostic testing needs to be available so that fish with uninflated swim bladders can be identified and removed. Additional research focusing on other risk factors for side swimming would also be very useful, given the < 100% PPV for swim bladders assessed to be abnormal in morphology or position.
